# Supplementary material for: TopDIA: A Software Tool for Top-Down Data-Independent Acquisition Proteomics
Source: J Proteome Res. 2024 Dec 6;24(1):55–64. doi: 10.1021/acs.jproteome.4c00293 (PMC11705214; doi:10.1021/acs.jproteome.4c00293)
Supplement: Supplementary file 1 — pr4c00293_si_001.pdf [file pr4c00293_si_001.pdf]

Supporting Information

TopDIA: A Software Tool for Top-Down Data-Independent Acquisition Proteomics

Abdul Rehman Basharat[1+], Xingzhao Xiong[2+], Tian Xu[3], Yong Zang[4], Liangliang Sun[3], and Xiaowen Liu[2,*]

[1]Department of BioHealth Informatics, School of Informatics and Computing, Indiana University-Purdue University Indianapolis, Indianapolis, IN, 46202, USA
[2]Deming Department of Medicine, Tulane University School of Medicine, New Orleans, LA, 70112, USA
[3]Department of Chemistry, Michigan State University, East Lansing, MI, 48824, USA
[4]Department of Biostatistics and Health Data Sciences, Indiana University School of Medicine, Indianapolis, IN, 46202, USA

[+]Co-first authors
*Correspondence: Xiaowen Liu (xwliu@tulane.edu); Liangliang Sun (lsun@chemistry.msu.edu)

Table of Content

Supplementary Figures...3

Supplementary Figure S1: A TD-DIA-MS experiment is divided into cycles, each of which contains an MS1 scan and 20 MS/MS scans. All the cycles in a run are sorted in the increasing order of the retention time and the index of a cycle is its position in the sorted list..3

Supplementary Figure S2: Extracted ion chromatograms (XICs) of an SCPF and a fragment feature are linear interpolated. Subsequently, the interpolated intensities within the XICs are normalized so that the area under the XIC equals 1. The shared area under the normalized interpolated XICs is reported as the shared XIC of the SCPF and the fragment feature. 3

Supplementary Figure S3: The receiver operating characteristic (ROC) curve of the logistic regression model on the test SCPF and fragment feature pairs generated from the DIA-TRAIN data. The AUC ROC of the model is 84.78%..........................4

Supplementary Figure S4: A histogram of the apex cycle distances of the positive SCPF fragment feature pairs in the DIA-TRAIN test data set. 4

Supplementary Figure S5: True positive and false positive rates of the logistic regression model with various score cutoff values on the SCPF and fragment feature pairs in the DIA-TRAIN test data set. 5

Supplementary Figure S6: Comparison of the S/N ratios (log-transformed, base 10) of the best single MS/MS spectrum and the average spectrum corresponding to the best pseudo spectrum for each of the 451 proteoforms identified from the DIA-TEST-1 data set by both the single spectra and pseudo spectra methods. 5

Supplementary Figure S7: Comparison of the deconvoluted fragment masses and matched b- and y-ion masses in the best single deconvoluted MS/MS spectra and the best pseudo spectra matched to the 451 proteoforms identified from the DIA-TEST-1 data set by both the single spectra and pseudo spectra methods. 6

Supplementary Figure S8: Distribution of the retention time ranges of all proteoforms features in the six runs of the DIA-TEST-1 data. 6

Supplementary Figure S9: The precursor features of proteoform P1 from UPF0339 protein YegP (UniProt ID: P76402, mass: 11,885.99 Da) and proteoform P2 from UPF0234 protein YajQ (UniProt ID: P0A8E7, mass: 3,393.63 Da) are observed in the isolation window [848-852] *m*/*z* of the DIA-TEST-1 data. The intensity of P1 is 4.5 times higher than that of P2 in DIA-TEST-1 and P2 is not identified due to the limited number of matched fragment ions in the multiplexed MS/MS spectra. 7

Supplementary Tables 8

Supplementary Table S1: Parameter settings for the modified version of TopFD for extracting proteoform features from TD-DIA-MS data 8

Supplementary Table S2: Parameter settings for the modified version of TopFD for extracting fragment features from TD-DIA-MS/MS data 8

Supplementary Table S3: Parameter settings for TopFD (version 1.7.2) 8

Supplementary Table S4: Variable PTMs used in TopPIC for protein identification 9

Supplementary Table S5: Parameter settings of TopPIC for analyzing TD-DDA-MS and TD-DIA-MS data sets 9

Supplementary Table S6: Comparison of proteoform and protein identifications in DIA-TRAIN with and without the third round of filtering in the generation of pseudo MS/MS spectra 9

Supplementary Table S7: Numbers of proteoform features and generated pseudo-MS/MS spectra with five or more fragment masses in the DIA-TEST-1 and DIA-TEST-2 data sets ...10

Supplementary Table S8: Numbers of proteoform features and MS/MS spectra in the DDA-TEST-1 and DDA-TEST-2 data sets 10

Supplementary Table S9: Comparison of proteoform and protein identifications between DIA-TEST-1 and DDA-TEST-1 10

Supplementary Table S10: Comparison of proteoform and protein identifications between DIA-TEST-2 and DDA-TEST-2 10

Supplementary Table S11: Comparison of proteoforms and proteins identified from DIA-TEST-1 by the pseudo spectra and single spectra approaches 11

Supplementary Table S12: Comparison of proteoform and protein identifications between DIA-TEST-1 and DIA-TEST-2 11

Supplementary Table S13: Comparison of proteoform and protein identifications between DDA-TEST-1 and DDA-TEST-2 11

Supplementary Note 12

Supplementary Note S1: TD-DDA-MS without gas fractionation 12

Supplementary Figures

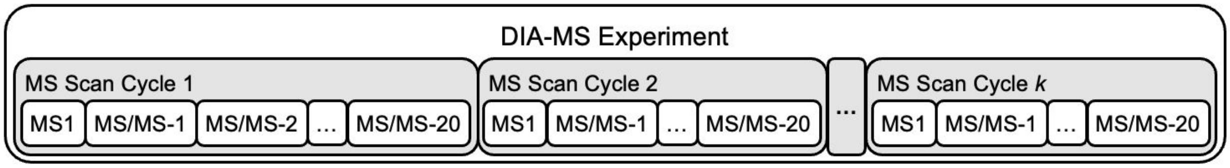

Supplementary Figure S1: A TD-DIA-MS experiment is divided into cycles, each of which contains an MS1 scan and 20 MS/MS scans. All the cycles in a run are sorted in the increasing order of the retention time and the index of a cycle is its position in the sorted list.

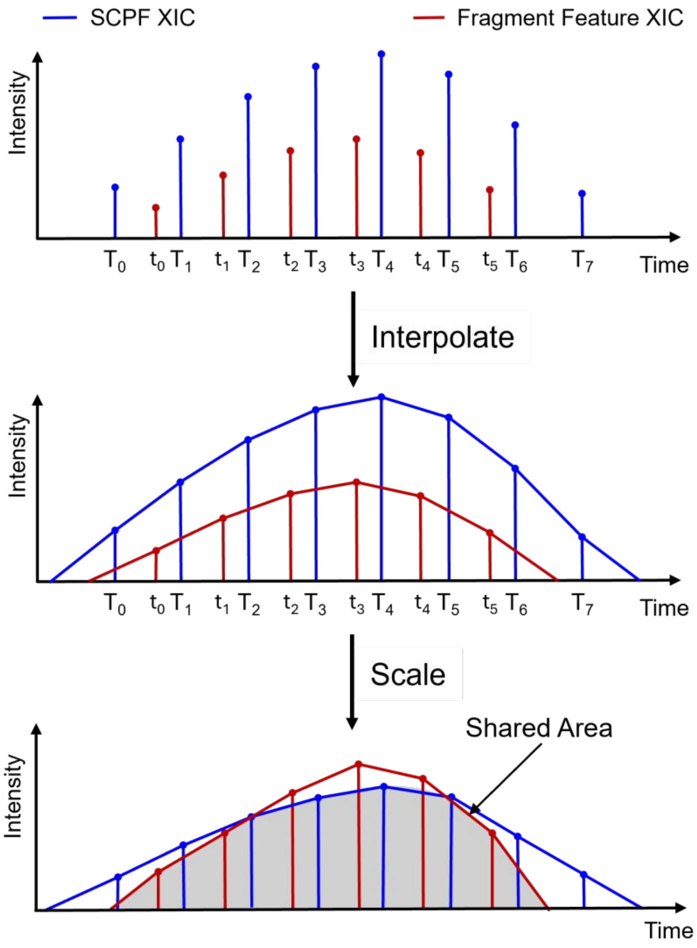

Supplementary Figure S2: Extracted ion chromatograms (XICs) of an SCPF and a fragment feature are linear interpolated. Subsequently, the interpolated intensities within the XICs are normalized so that the area under the XIC equals 1. The shared area under the normalized interpolated XICs is reported as the shared XIC of the SCPF and the fragment feature.

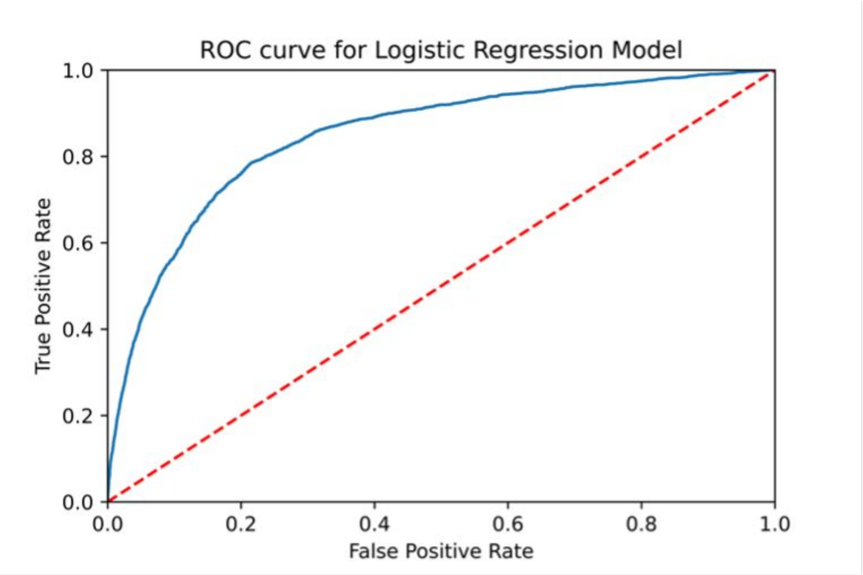

Supplementary Figure S3: The receiver operating characteristic (ROC) curve of the logistic regression model on the test SCPF and fragment feature pairs generated from the DIA-TRAIN data. The AUC ROC of the model is 84.78%.

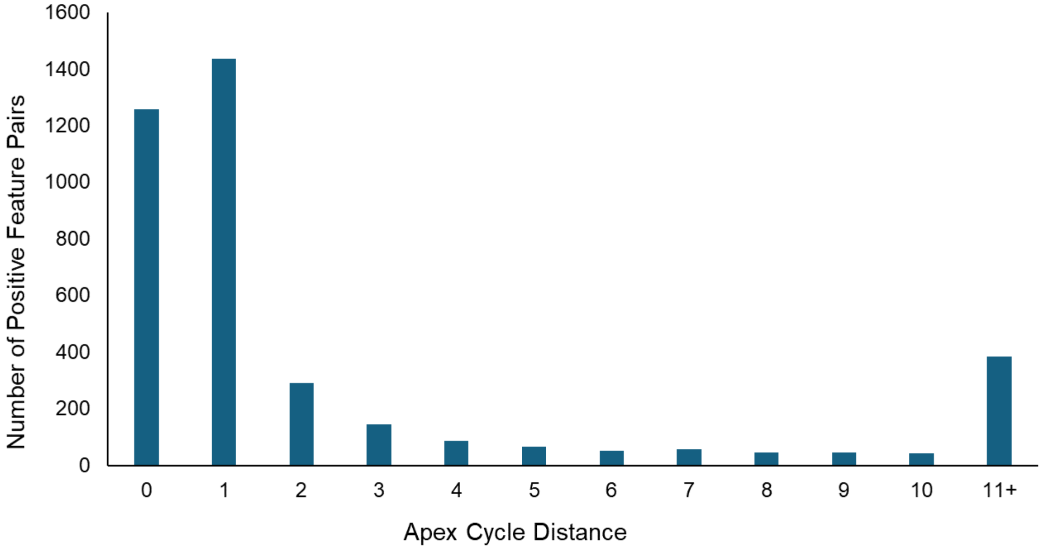

Supplementary Figure S4: A histogram of the apex cycle distances of the positive SCPF fragment feature pairs in the DIA-TRAIN test data set.

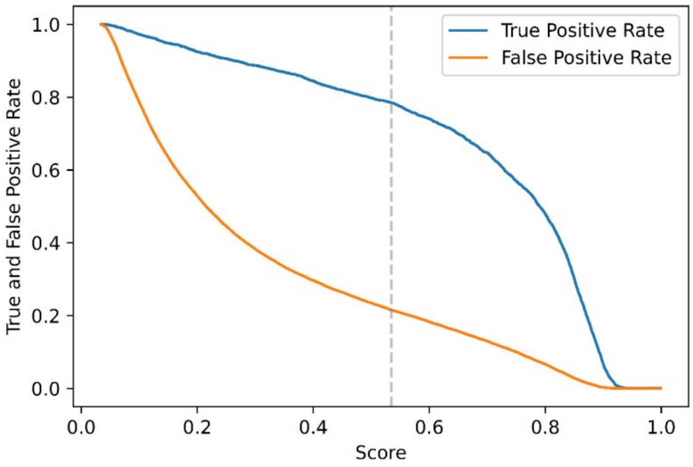

Supplementary Figure S5: True positive and false positive rates of the logistic regression model with various score cutoff values on the SCPF and fragment feature pairs in the DIA-TRAIN test data set.

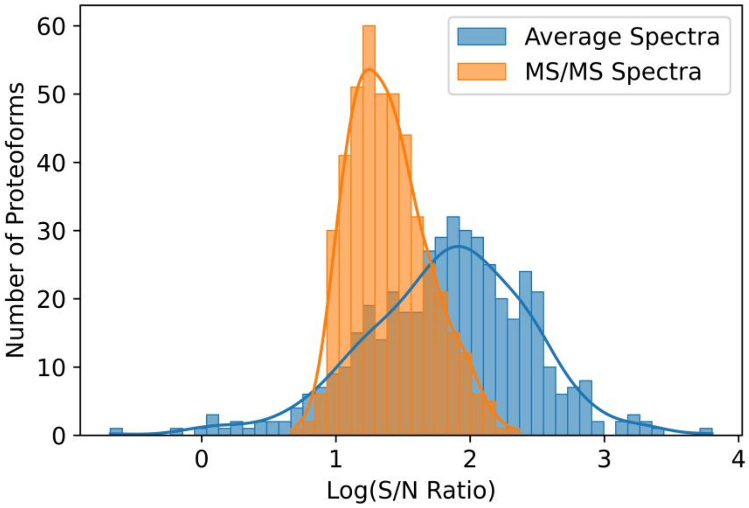

Supplementary Figure S6: Comparison of the S/N ratios (log-transformed, base 10) of the best single MS/MS spectrum and the average spectrum corresponding to the best pseudo spectrum for each of the 451 proteoforms identified from the DIA-TEST-1 data set by both the single spectra and pseudo spectra methods.

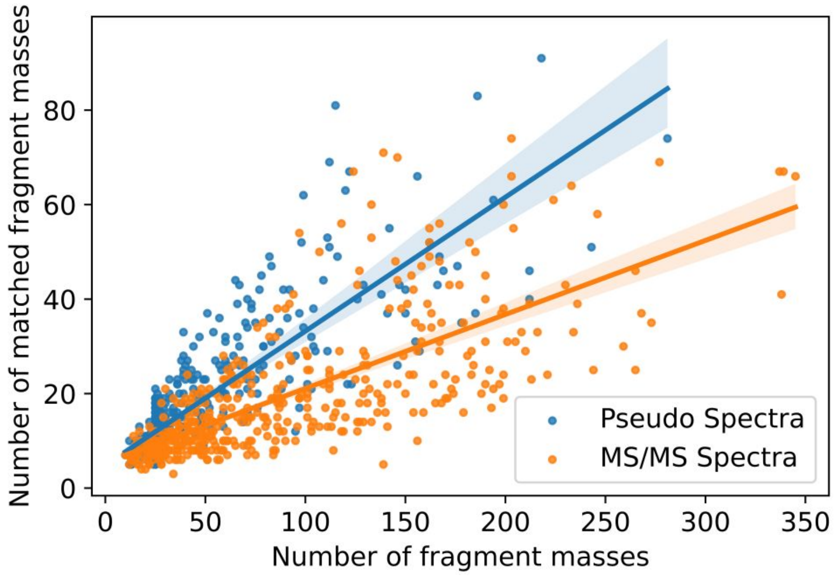

Supplementary Figure S7: Comparison of the deconvoluted fragment masses and matched b- and y-ion masses in the best single deconvoluted MS/MS spectra and the best pseudo spectra matched to the 451 proteoforms identified from the DIA-TEST-1 data set by both the single spectra and pseudo spectra methods.

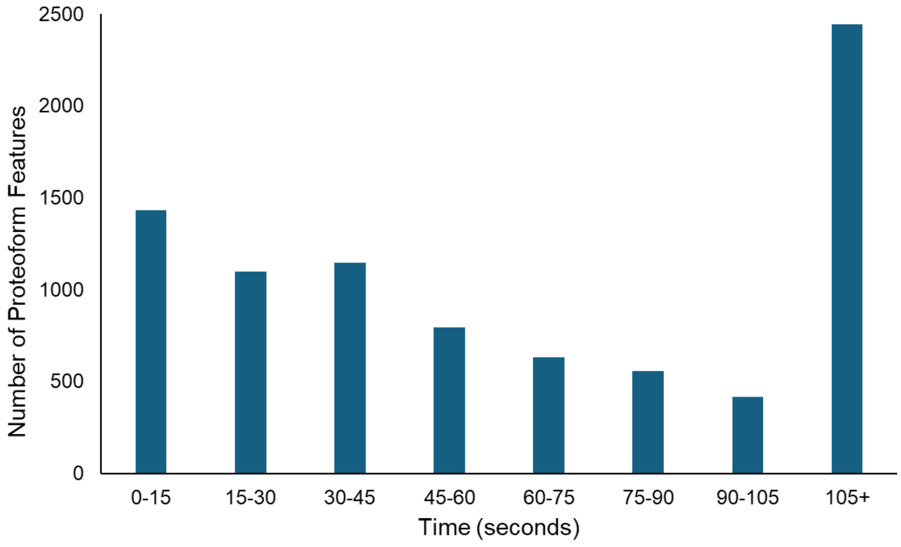

Supplementary Figure S8: Distribution of the retention time ranges of all proteoforms features in the six runs of the DIA-TEST-1 data.

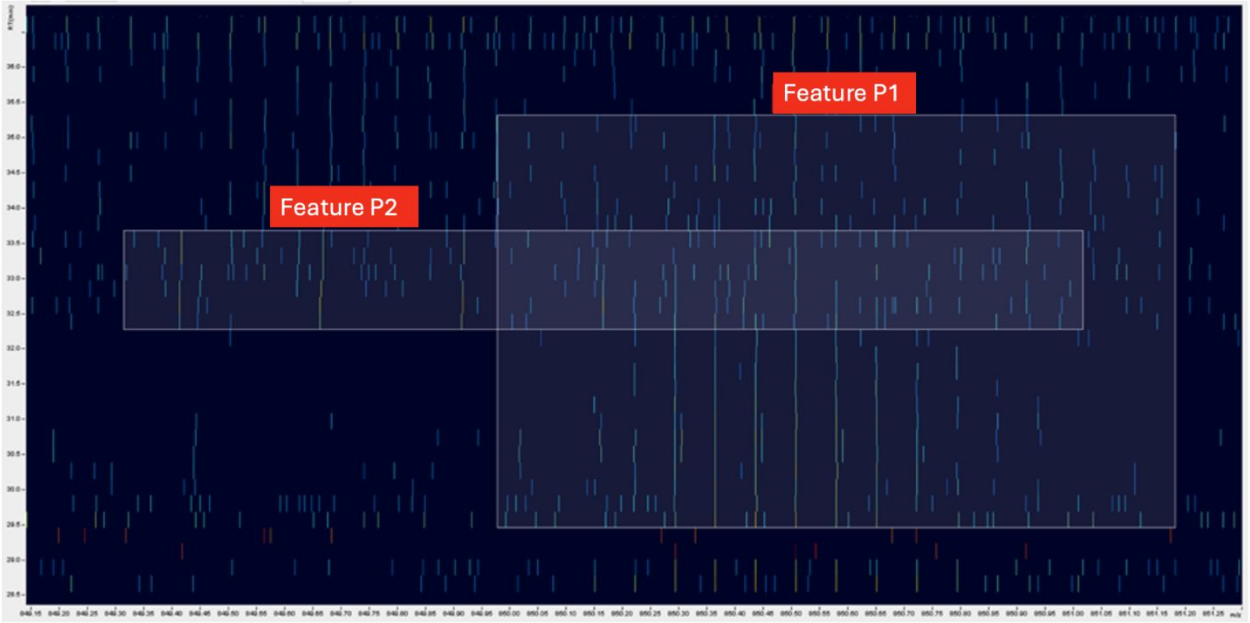

Supplementary Figure S9: The precursor features of proteoform P1 from UPF0339 protein YegP (UniProt ID: P76402, mass: 11,885.99 Da) and proteoform P2 from UPF0234 protein YajQ (UniProt ID: P0A8E7, mass: 3,393.63 Da) are observed in the isolation window [848-852] *m*/*z* of the DIA-TEST-1 data. The intensity of P1 is 4.5 times higher than that of P2 in DIA-TEST-1 and P2 is not identified due to the limited number of matched fragment ions in the multiplexed MS/MS spectra.

Supplementary Tables

Supplementary Table S1: Parameter settings for the modified version of TopFD for extracting proteoform features from TD-DIA-MS data

Parameter	Value
Maximum charge	60
Maximum mass	70,000 Da
MS1 signal noise ratio in MS-Deconv	3.0
M/z error tolerance in MS-Deconv	0.02
Do final filtering in MS-Deconv	True
Use EnvCNN score in MS-Deconv	True
Use single scan noise level during feature extraction	True
Minimum scan number in features	2
Seed envelope intensity correlation tolerance	0.5
ECScore cutoff	0

Supplementary Table S2: Parameter settings for the modified version of TopFD for extracting fragment features from TD-DIA-MS/MS data

Parameter	Value
Maximum charge	60
Maximum mass	70,000 Da
MS/MS signal noise ratio in MS-Deconv	1.0
Isolation window	4.0 *m/z*
M/z error tolerance in MS-Deconv	0.02
Do final filtering in MS-Deconv	True
Use single scan noise level during feature extraction	True
Minimum scan number in features	1
Seed envelope intensity correlation tolerance	0
ECScore cutoff	0

Supplementary Table S3: Parameter settings for TopFD (version 1.7.2)

Parameter	Value
Maximum charge	60
Maximum mass	70,000 Da
MS1 signal noise ratio in MS-Deconv	3.0
MS/MS signal noise ratio in MS-Deconv	1.0
M/z error tolerance in MS-Deconv	0.02
Do final filtering in MS-Deconv	True
Use EnvCNN score in MS-Deconv	True
Use single scan noise level during feature extraction	True
Isolation window	DDA: 3.0 *m/z*<br>DIA: 4.0 *m/z*
Minimum scan number in features	DDA: 3<br>DIA: 2
Seed envelope intensity correlation tolerance	0.5
ECScore cutoff	0.5
Additional feature search	False

Supplementary Table S4: Variable PTMs used in TopPIC for protein identification

PTM	Mass (Da)	Possible amino acid sites
Methylation	14.015650	CKRHDENQ
Oxidation	15.994915	CPKDNRY
Acetylation	42.010565	Any
Phosphorylation	79.966331	Any

Supplementary Table S5: Parameter settings of TopPIC for analyzing TD-DDA-MS and TD-DIA-MS data sets

Parameter	Value
Fragmentation method	File
Search type	Target+Decoy
N-terminal forms of proteins	NONE, M_ACETYLATION, NME, NME_ACETYLATION
Fixed modifications	No
Maximum number of variable modifications	3
Variable modifications	Methylation, Oxidation, Acetylation, and Phosphorylation
Spectrum level cutoff type for filtering PrSMs	FDR
The cutoff value for filtering PrSMs	0.01
Spectrum level cutoff type for filtering proteoforms	FDR
The cutoff value for filtering proteoforms	0.01
Error tolerance for precursor and fragment masses	10 ppm
Error tolerance for identifying PrSM clusters	1.2 Da
Maximum number of unexpected mass shifts	1
Minimum value of the mass shift	-500 Da
Maximum value of the mass shift	500 Da
E-values computation	Generating function
Use TopFD feature	DDA: True<br>DIA: False

Supplementary Table S6: Comparison of proteoform and protein identifications in DIA-TRAIN with and without the third round of filtering in the generation of pseudo MS/MS spectra

m/z range	With Filter		Without Filter	
	Proteoforms	Proteins	Proteoforms	Proteins
720-800	180	85	178	85
800-880	186	88	186	87
880-960	182	93	181	93
960-1040	227	102	226	102
1040-1120	193	90	193	90
1120-1200	161	80	161	80

Supplementary Table S7: Numbers of proteoform features and generated pseudo-MS/MS spectra with five or more fragment masses in the DIA-TEST-1 and DIA-TEST-2 data sets

m/z range	DIA-TEST-1		DIA-TEST-2	
	Pseudo Spectra	Proteoform Features	Pseudo Spectra	Proteoform Features
720-800	937	1244	841	1156
800-880	1107	1459	948	1253
880-960	1319	1577	1287	1523
960-1040	1055	1356	1086	1357
1040-1120	1150	1451	1137	1369
1120-1200	973	1432	899	1320

Supplementary Table S8: Numbers of proteoform features and MS/MS spectra in the DDA-TEST-1 and DDA-TEST-2 data sets

m/z range	DDA-TEST-1		DDA-TEST-2	
	MS/MS Spectra	Proteoform Features	MS/MS Spectra	Proteoform Features
720-800	7556	1174	7550	1083
800-880	7628	1131	7626	1076
880-960	7614	1239	7614	1162
960-1040	7587	1147	7608	1099
1040-1120	7552	1249	7569	1180
1120-1200	7495	1280	7515	1208

Supplementary Table S9: Comparison of proteoform and protein identifications between DIA-TEST-1 and DDA-TEST-1

m/z range	DIA		DDA		Shared	
	Proteoforms	Proteins	Proteoforms	Proteins	Proteoforms	Proteins
720-800	176	84	173	82	122	72
800-880	199	93	209	91	134	78
880-960	205	98	263	109	164	88
960-1040	202	95	233	101	151	84
1040-1120	192	92	190	77	136	68
1120-1200	166	78	157	73	113	60

Supplementary Table S10: Comparison of proteoform and protein identifications between DIA-TEST-2 and DDA-TEST-2

m/z range	DIA		DDA		Shared	
	Proteoforms	Proteins	Proteoforms	Proteins	Proteoforms	Proteins
720-800	151	72	171	74	108	62
800-880	210	94	175	75	126	67
880-960	213	103	228	94	152	81
960-1040	217	105	225	99	145	80
1040-1120	201	97	226	97	148	79
1120-1200	134	70	178	79	106	59

Supplementary Table S11: Comparison of proteoforms and proteins identified from DIA-TEST-1 by the pseudo spectra and single spectra approaches

m/z range	Pseudo spectra		Single spectra		Shared	
	Proteoforms	Proteins	Proteoforms	Proteins	Proteoforms	Proteins
720-800	176	84	167	76	130	72
800-880	199	93	182	82	144	78
880-960	205	98	211	90	154	83
960-1040	202	95	187	91	153	85
1040-1120	192	92	159	73	134	71
1120-1200	166	78	155	68	126	63

Supplementary Table S12: Comparison of proteoform and protein identifications between DIA-TEST-1 and DIA-TEST-2

m/z range	DIA-TEST-1		DIA-TEST-2		Shared	
	Proteoforms	Proteins	Proteoforms	Proteins	Proteoforms	Proteins
720-800	176	84	151	72	109 (72.2%)	64 (88.9%)
800-880	199	93	210	94	126 (72.0%)	75 (89.3%)
880-960	205	98	213	103	145 (70.7%)	84 (85.7%)
960-1040	202	95	217	105	143 (70.8%)	83 (87.4%)
1040-1120	192	92	201	97	141 (74.2%)	82 (89.1%)
1120-1200	166	78	134	70	104 (77.6%)	63 (90.0%)

Supplementary Table S13: Comparison of proteoform and protein identifications between DDA-TEST-1 and DDA-TEST-2

m/z range	DDA-TEST-1		DDA-TEST-2		Shared	
	Proteoforms	Proteins	Proteoforms	Proteins	Proteoforms	Proteins
720-800	173	82	171	74	115 (76.1%)	66 (94.3%)
800-880	209	91	175	75	145 (82.8%)	73 (97.3%)
880-960	263	109	228	94	161 (78.5%)	80 (86.9%)
960-1040	233	101	225	99	160 (79.2%)	84 (92.3%)
1040-1120	190	77	226	97	158 (83.1%)	74 (96.1%)
1120-1200	157	73	178	79	113 (84.3%)	62 (93.9%)

Supplementary Note

Supplementary Note S1: TD-DDA-MS without gas fractionation

We conducted a TD-DDA-MS analysis of an *E. coli* sample using a single run with an *m*/*z* range of [720, 1200]. A 188-min gradient of mobile phase B (0-5 min 5%, 5-7 min for 5% to 35%, 7-15 min for 35% to 50%, 15-187 min for 50% to 80%, 187-188 min from 80% to 99%) was applied with a flow rate of 400 nL/min in the LC separation. Other experimental parameters in the LC-MS experiment were the same as the TD-DDA-MS experiments described in section Top-down RPLC-MS/MS.

TopFD was used to analyze the 188-min separation *E. coli* TD-DDA-MS data set for proteoform feature detection and spectral deconvolution. The parameter settings of TopFD are given in Supplementary Table S3. Using TopPIC, we identified 309 proteoforms from 138 proteins with a 1% proteoform-level FDR in the single-run experiment. The parameter settings of TopPIC are given in Supplementary Table S5. Compared with the single-run TD-DDA-MS data, the six-run TD-DDA-MS approach increased proteoform identifications from 309 to 574 and protein identifications from 138 to 186 (see section Results).
